# Proximity to Legal Cannabis Stores in Canada and Use of Cannabis Sources in the First Three Years of Legalization, 2019–2021

**DOI:** 10.15288/jsad.22-00427

**Published:** 2023-11-08

**Authors:** Elle Wadsworth, Pete Driezen, Julia A. Dilley, Robert Gabrys, Rebecca Jesseman, David Hammond

**Affiliations:** ^a^ School of Public Health Sciences, University of Waterloo, Waterloo, Ontario, Canada; ^b^ Canadian Centre on Substance Use and Addiction, Ottawa, Ontario, Canada; ^c^ Department of Psychology, University of Waterloo, Waterloo, Ontario, Canada; ^d^ Program Design and Evaluation Services, Multnomah County Health Department and Oregon Health Authority Division of Public Health, Portland, Oregon; ^e^ Mental Health and Addictions, Health PEI, Charlottetown, Prince Edward Island, Canada

## Abstract

**Objective:**

The accessibility of legal cannabis in Canada may influence how consumers source their cannabis. The aims of this study were to examine (a) the distance between respondents’ homes and legal retail stores, (b) the cannabis sources used in the past 12 months, and (c) the association between cannabis sources used and distance to legal retail stores.

**Method:**

Data were analyzed from Canadian respondents participating in the International Cannabis Policy Study from 2019 to 2021. Respondents were 15,311 past-12-month cannabis consumers of legal age to purchase cannabis. Weighted logistic regression models examined cannabis sources used and their association with the Euclidean distance to the nearest legal store, province of residence, and year (*n* = 12,928).

**Results:**

Respondents lived closer to a legal retail store in 2021 (1.5 km) versus 2019 (6.8 km) as the number of retail stores increased. Respondents in 2020 and 2021 had higher odds of obtaining cannabis from legal sources (e.g., legal stores: 47.9% and 60.0% vs. 38.6%, respectively, adjusted odds ratio [AOR] range: 1.41–2.42) and lower odds of obtaining cannabis from illegal sources versus 2019 (e.g., dealers: 22.6% and 19.9% vs. 29.1%, respectively, AOR range: 0.65–0.54). Respondents who lived closest to legal stores had higher odds of sourcing from legal stores and lower odds of sourcing from legal websites or growing their own cannabis.

**Conclusions:**

Legal cannabis stores are increasingly accessible to people living in Canada 3 years after legalization. Household proximity to a legal cannabis store was associated with sourcing cannabis from legal retail stores, but only among those who live very close (<3 km). Findings suggest that proximity to legal cannabis stores may aid uptake of the legal market, yet there may be diminishing returns after a certain point.

In October 2018, Canada legalized nonmedical (“recreational”) cannabis. The Cannabis Act not only legalized nonmedical cannabis but also replaced the previous regulations for medical cannabis, which has been legally available since 2001. Along with removing penalties for consumption, legalization in Canada introduced a legal retail market, allowing adult consumers to purchase cannabis products from physical retail stores and online stores and to grow their own cannabis at home (except in two provinces: Québec and Manitoba). Before legalization in 2018, prevalence of past-12-month cannabis use was 22% and since legalization has increased to 27% in 2022 (Government of Canada, 2022). Among past-12-month cannabis consumers, daily or almost daily consumption has remained unchanged from 2018 to 2022 (25%; Government of Canada, 2022).

Cannabis legalization in Canada was enacted at the federal level; however, all 10 provinces and 3 territories have jurisdiction over certain regulatory decisions, such as minimum legal age, retail structure (private, government run, or hybrid), and the number and location of legal retail stores. For instance, Alberta has a private retail structure and had 761 stores as of September 2022. In contrast, Québec has a government-run retail structure and had 90 stores as of September 2022. As Québec's retail store locations are chosen by the government rather than the market, they are geographically more diverse and have been centrally planned to match population distribution (Gibbs et al., 2021). Further research is needed to determine whether access and proximity to retail stores across provinces influence which cannabis source consumers use.

Several Canadian provinces allow municipalities to prohibit legal cannabis stores from operating within their boundaries (i.e., “opt out”). Such prohibitions may have restricted access to legal cannabis stores. For example, in Ontario, several large cities in the Greater Toronto Area have opted out of retail stores (Alcohol and Gaming Commission of Ontario, 2022). Local opt out has also been allowed in the United States by states that have legalized cannabis markets (Department of Cannabis Control, 2022; Dilley et al., 2017). For example, in California, 61% of cities and counties prohibit cannabis retail stores as of September 2022; however, these residents can access legal cannabis through delivery services (Department of Cannabis Control, 2022). Local opt out regulations may influence the relative ease of access between legal and illegal sources.

Distribution of legal stores may plausibly affect how people obtain cannabis and whether they transition to using legal sources. Limited access to “brick-and-mortar” stores may increase the likelihood that consumers grow their own cannabis. Previous research has demonstrated that respondents living in rural areas were more likely to grow their own cannabis than those living in urban settings, perhaps because of reduced access to alternative cannabis sources (Azofeifa et al., 2021; Wadsworth et al., 2022). The availability of online legal purchases may also influence the relative importance of geographic proximity to brick-and-mortar stores. The COVID-19 pandemic affected access to many services; however, to ensure access to legal cannabis throughout periods during which physical retail stores were closed (i.e., “lock-downs”), provinces expanded online access and introduced curbside pickup and same-day delivery in some areas (Canadian Centre on Substance Use and Addiction, 2021; Leafly Canada Staff, 2019). Indeed, research using government sales data concluded that the pandemic had minimal impact on legal cannabis sales in Canada (Armstrong et al., 2022).

Previous research examining proximity to cannabis stores (medical and nonmedical) has primarily focused on its association with cannabis consumption among U.S. adults (Everson et al., 2019) and among youth (Firth et al., 2022; Hust et al., 2020; Palali & van Ours, 2015; Shi, 2016; Shi et al., 2018). Indeed, prior research from tobacco and alcohol indicates that increased retail availability (either proximity or retail density) is associated with increased initiation and prevalence of use, especially among youth (Freisthler et al., 2003; Lipperman-Kreda et al., 2012, 2014). However, for proximity to cannabis retail stores and consumption, the findings are mixed: some studies conclude that there is an association between proximity and increased use (Everson et al., 2019; Firth et al., 2022; Hust et al., 2020; Palali & van Ours, 2015), whereas other studies report no association (Firth et al., 2022; Shi, 2016; Shi et al., 2018). Other research has examined the association between legal retail stores and legal sales in the Canadian market (Armstrong, 2021; Armstrong et al., 2022). Using Canadian government data from the first 3 years of legalization, Armstrong (2021) found that the expansion of the cannabis retail market through increased retail stores was positively associated with legal sales growth.

Understanding the impact of retail proximity on the likelihood of sourcing legally has important implications for cannabis policy. Consumer access to legal stores is an important factor in displacing the illegal market; however, high levels of retail density also have the potential to promote more frequent consumption (Everson et al., 2019; Hust et al., 2020; Popova et al., 2009). Sourcing from the legal market has benefits to the consumer including access to cannabis of known composition and potency that is labeled according to government standards and that is subject to product safety testing. Illegal products are not subject to any of these health and safety protections. Therefore, jurisdictions with legal markets should determine the balance of providing accessible retail to displace the illegal market, without encouraging more frequent consumption or initiation. This study had three aims, as follows: (a) to estimate the average distance to the nearest legal retail store across the provinces among past-12-month cannabis consumers of provincial legal age to purchase cannabis, (b) to examine the cannabis sources used across distance to the nearest legal retail store, and (c) to examine the association between cannabis sources used and distance to the nearest legal retail store in 2019–2021.

## Method

Data were from Waves 2–4 of the International Cannabis Policy Study (ICPS), which consists of repeat cross-sectional surveys. The current study reports data from the Canadian ICPS sample only. Data were collected via self-completed web-based surveys in September–October in 2019, 2020, and 2021 from respondents ages 16–65 years. A nonprobability sample of respondents was recruited through the Nielsen Consumer Insights Global Panel and their partners’ panels. For the ICPS surveys, Nielsen draws stratified random samples from the online panels, with quotas based on age and province of residence. Quotas are also used for recruiting sample size proportional to the population size of the provinces, and poststratification weights are used (see *Statistical analysis* section). Nielsen emailed panelists an invitation to access the ICPS survey via a unique link; respondents were unaware of the survey topic before accessing the link. Respondents confirmed their eligibility and provided consent before completing the survey. Surveys were conducted in English or French. Median survey time was 25 minutes in 2019, 21 minutes in 2020, and 22 minutes in 2021. Upon completion, respondents received remuneration.

The study was reviewed by and received ethics clearance through a University of Waterloo Research Ethics Committee (ORE#31330). A full description of the study methods can be found in the ICPS Technical Reports and methodology paper (Corsetti et al., 2022; Goodman et al., 2020, 2021; Hammond et al., 2020).

### Measures

*Sociodemographic measures*. Sex at birth, age, ethnicity/ race, highest education level, perceived income adequacy, device type used to complete survey, and province of residence were measured. *Perceived income adequacy* was defined as “the perceived ability to make ends meet.” Minimum legal age to purchase cannabis (MLA) was taken from provincial laws in September 2019, 2020, and 2021. MLA in Alberta was 18 in all years; MLA in Québec was 18 in 2019 and 21 in 2020 and 2021; and MLA in all other provinces was 19 in all years. Québec was chosen as the reference category in regression models because it had the lowest number of stores per capita in all 3 years (Canadian Centre on Substance Use and Addiction, 2022; Myran et al., 2022; Wadsworth et al., 2021). For perceived income adequacy and highest level of education, those who answered *don't know* or *refuse to answer* were categorized as *not stated*.

*Cannabis use frequency*. Cannabis use frequency was assessed using two questions: “How often do you use cannabis?” and “When was the last time you used cannabis?” Responses were categorized as *less than monthly consumer but used in the past 12 months, monthly consumer, weekly consumer*, and *daily consumer (daily or almost daily consumer)*.

*Cannabis source used in the past 12 months*. Respondents were asked, “In the past 12 months, have you gotten any type of cannabis from the following sources?” with the following response options: I made or grew my own, *from a family member or friend, from a dealer (in person), internet delivery service or mail order (delivered to me), from a store, co-operative or dispensary (in person/curbside pickup)*, and *other*. Respondents could select all that applied. For the remainder of the manuscript, *I made or grew my own* is referred to as *grow their own*. For physical and online retail stores, the legality of these sources was determined by follow-up questions, “What type of [physical store or dispensary/online source] did you buy cannabis in the past 12 months?” Response options were *a legal/authorized [store/ website], an illegal or unauthorized [store or dispensary/ website, private delivery service or dealer]*, and *other*. Respondents could select all that applied.

In total, the following seven binary outcomes were created: (a) Grew their own, (b) Friends or family, (c) Dealer in person, (d) Legal stores, (e) Illegal stores or dispensaries, (f) Legal websites, and (g) Illegal websites. Binary responses were *yes* or *no*.

*Household proximity (Euclidean distance to nearest legal retail store)*. Respondents were asked, “Please provide the postal code where you live for most of the year.” A total of 2,900 respondents in 2019, 2,934 in 2020, and 3,032 in 2021 either didn't know their postal code, refused to answer, or had a postal code that did not match their province of residence (*n*_2019_ = 2,900; *n*_2020_ = 2,934; *n*_2021_ = 3,032). If respondents answered *don't know* or *refuse to answer*, they were asked, “Instead of providing your postal code, would you feel comfortable telling us the nearest intersection to your home? Please name the 2 cross-streets of this intersection.” Of those who provided their intersection (*n*_2019_ = 1,081; *n*_2020_ = 1,068; *n*_2021_ = 1,063), Google Maps was used to obtain postal codes, cross-referencing with the respondent's city and province. All intersections for which Google Maps could not find a postal code were left blank (*n*_2019_ = 272; *n*_2020_ = 336; *n*_2021_ = 306). A total of 809 postal codes in 2019, 732 in 2020, and 757 in 2021 were retrieved. In the final data set, 84.8% of respondents in 2019, 85.8% in 2020, and 86.3% in 2021 included postal code information.

Legal retailers and their postal codes in each province were identified from provincial websites in September of each year and cross-checked with lists displayed on Leafly (www.leafly.ca). Illegal retail stores were not included. The Canadian respondents’ postal codes and postal codes of legal retail stores were then linked to the Postal Code Conversion File Plus (PCCF+) Version 7B, 7C, or 7D, to obtain latitude and longitudes (Statistics Canada, 2021). An open-source geographic information system application (QGIS Version 3.6; qgis.org) was used to geocode the latitudes and longitudes, and the Euclidean distance (in kilometers) between the centroid of the postal code of each respondent's address and their nearest legal retail store was computed. In urban areas, Canadian postal codes can cover a single house/apartment building, whereas postal codes in rural areas cover a larger area and a centroid is used. In the current study, 86% (2019, 2020) to 87% (2021) of respondents lived in urban areas; therefore, a certain degree of accuracy can be assumed from postal codes as a proxy for respondents’ residence (Bow, 2004; Pinault et al., 2020). The North American Equidistant Conic Projection (EPSG:102010) was used to minimize distance distortions. Distances were categorized as *under 3 km, 3–4.99 km, 5–9.99 km*, or *over 10 km* to mimic categories used by Statistics Canada (2019). A sensitivity analysis was conducted in which those who did not provide a postal code were included as a separate category (Supplemental Table A). (Supplemental material appears as an online-only addendum to this article on the journal's website.)

*Rural/urban*. For respondents who provided a postal code, their postal code was assigned *rural* or *urban* from the PCCF+ Version 7B, 7C, or 7D (Statistics Canada, 2021).

All questions included *don't know* and *refuse to answer* options. All *don't know* and *refuse to answer* options were excluded unless specified within the measures above.

### Analytic sample

Among Canadian respondents, the survey had an American Association for Public Opinion Research (AAPOR) cooperation rate of 63% in 2019, 66% in 2020, and 68% in 2021 (AAPOR, 2016). The cooperation rate is “the number of respondents who have provided a usable response divided by the total number of initial personal invitations requesting participation” (AAPOR, 2016). The response rate was 2.1% in 2019, 1.1% in 2020, and 1.3% in 2021. The final Canadian cross-sectional samples comprised 15,256 respondents in 2019; 15,780 in 2020; and 16,952 in 2021. See Technical Reports for more details (Corsetti et al., 2022; Goodman et al., 2020, 2021).

Analyses were conducted on the subsample of respondents who had consumed cannabis in the past 12 months and were of provincial legal age to purchase cannabis products (*n*_2019_ = 4,857; *n*_2020_ = 4,652; *n*_2021_ = 5,802). Missing data were removed using casewise deletion for variables used in regression models: highest level of education (*n* = 125 [0.8%]); perceived income adequacy (*n* = 344 [2.2%]); and Euclidean distance to the nearest legal retail store (*n* = 2,249 [14.7%]).

### Statistical analysis

Poststratification sample weights were constructed based on the 2016 Canadian Census estimates. Respondents were classified into age-by-sex-by-province, education, and ageby-tobacco cigarette status groups. For Canada, the percent change in the smoking rate from the Community Health Survey was used to determine the smoking rate for the survey weights (Statistics Canada, 2022). A raking algorithm was applied to the cross-sectional analytic samples to compute weights that were calibrated to these groupings (age-by-sexby-province, education, and age-by-tobacco cigarette status). The SAS macro “RAKE_AND_TRIM_G4_V5”17 was used, with trimming to 5 (rescaled). Weights were rescaled to the sample size for all years in Canada. Estimates are weighted unless otherwise specified.

First, the average Euclidean distance to the nearest legal retail store in kilometers was examined across province of residence and year. Second, the cannabis sources used in the past 12 months to obtain cannabis were examined in 2019–2021. Third, seven multivariable logistic regression models were fitted to examine past-12-month use of cannabis sources (dependent variable) and their association with retail proximity, province, and year (independent variables). All models were estimated with retail proximity as a categorical variable. Sensitivity analyses were conducted in which retail proximity included those who did not provide a postal code (Supplemental Table A). In all models, two-way interactions were conducted for survey wave and retail proximity to examine whether retail proximity changed over time as more stores opened in Canada. All models were adjusted for age, sex at birth, education level, ethnicity/race, income adequacy, and survey device type. Note that the “rural/urban” variable was not included in regression analysis because of multicollinearity with household proximity and province of residence. Adjusted odds ratios (AORs) are reported with 95% confidence intervals (95% CI). Analyses were conducted using survey procedures in SAS Version 9.4 (SAS Institute Inc., Cary, NC).

## Results

Table 1 displays the unweighted and weighted sample characteristics of respondents in the study sample. Across all years, close to half of respondents were male, three quarters identified as White ethnicity/race, and one third consumed cannabis daily/almost daily.

**Table 1.**
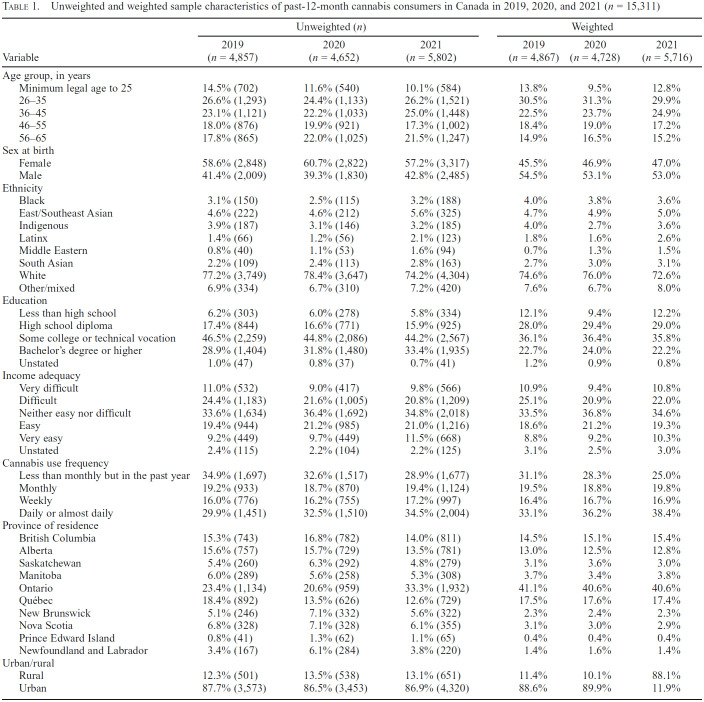
Unweighted and weighted sample characteristics of past-12-month cannabis consumers in Canada in 2019, 2020, and 2021 (*n* = 15,311)

Variable	Unweighted (*n*)			Weighted		
	2019	2020	2021	2019	2020	2021
Variable	(*n =* 4,857)	(*n* = 4,652)	(*n* = 5,802)	*(n =* 4,867)	(*n* = 4,728)	(*n =* 5,716)
Age group, in years						
Minimum legal age to 25	14.5% (702)	11.6% (540)	10.1% (584)	13.8%	9.5%	12.8%
26-35	26.6% (1,293)	24.4% (1,133)	26.2% (1,521)	30.5%	31.3%	29.9%
36-45	23.1% (1,121)	22.2% (1,033)	25.0% (1,448)	22.5%	23.7%	24.9%
46-55	18.0% (876)	19.9% (921)	17.3% (1,002)	18.4%	19.0%	17.2%
56-65	17.8% (865)	22.0% (1,025)	21.5% (1,247)	14.9%	16.5%	15.2%
Sex at birth						
Female	58.6% (2,848)	60.7% (2,822)	57.2% (3,317)	45.5%	46.9%	47.0%
Male	41.4% (2,009)	39.3% (1,830)	42.8% (2,485)	54.5%	53.1%	53.0%
Ethnicity						
Black	3.1% (150)	2.5% (115)	3.2% (188)	4.0%	3.8%	3.6%
East/Southeast Asian	4.6% (222)	4.6% (212)	5.6% (325)	4.7%	4.9%	5.0%
Indigenous	3.9% (187)	3.1% (146)	3.2% (185)	4.0%	2.7%	3.6%
Latinx	1.4% (66)	1.2% (56)	2.1% (123)	1.8%	1.6%	2.6%
Middle Eastern	0.8% (40)	1.1% (53)	1.6% (94)	0.7%	1.3%	1.5%
South Asian	2.2% (109)	2.4% (113)	2.8% (163)	2.7%	3.0%	3.1%
White	77.2% (3,749)	78.4% (3,647)	74.2% (4,304)	74.6%	76.0%	72.6%
Other/mixed	6.9% (334)	6.7% (310)	7.2% (420)	7.6%	6.7%	8.0%
Education						
Less than high school	6.2% (303)	6.0% (278)	5.8% (334)	12.1%	9.4%	12.2%
High school diploma	17.4% (844)	16.6% (771)	15.9% (925)	28.0%	29.4%	29.0%
Some college or technical vocation	46.5% (2,259)	44.8% (2,086)	44.2% (2,567)	36.1%	36.4%	35.8%
Bachelor's degree or higher	28.9% (1,404)	31.8% (1,480)	33.4% (1,935)	22.7%	24.0%	22.2%
Unstated	1.0% (47)	0.8% (37)	0.7% (41)	1.2%	0.9%	0.8%
Income adequacy						
Very difficult	11.0% (532)	9.0% (417)	9.8% (566)	10.9%	9.4%	10.8%
Difficult	24.4% (1,183)	21.6% (1,005)	20.8% (1,209)	25.1%	20.9%	22.0%
Neither easy nor difficult	33.6% (1,634)	36.4% (1,692)	34.8% (2,018)	33.5%	36.8%	34.6%
Easy	19.4% (944)	21.2% (985)	21.0% (1,216)	18.6%	21.2%	19.3%
Very easy	9.2% (449)	9.7% (449)	11.5% (668)	8.8%	9.2%	10.3%
Unstated	2.4% (115)	2.2% (104)	2.2% (125)	3.1%	2.5%	3.0%
Cannabis use frequency						
Less than monthly but in the past year	34.9% (1,697)	32.6% (1,517)	28.9% (1,677)	31.1%	28.3%	25.0%
Monthly	19.2% (933)	18.7% (870)	19.4% (1,124)	19.5%	18.8%	19.8%
Weekly	16.0% (776)	16.2% (755)	17.2% (997)	16.4%	16.7%	16.9%
Daily or almost daily	29.9% (1,451)	32.5% (1,510)	34.5% (2,004)	33.1%	36.2%	38.4%
Province of residence						
British Columbia	15 3% (743)	16.8% (782)	14.0% (811)	14.5%	15.1%	15.4%
Alberta	15.6% (757)	15.7% (729)	13.5% (781)	13.0%	12.5%	12.8%
Saskatchewan	5.4% (260)	6.3% (292)	4.8% (279)	3.1%	3.6%	3.0%
Manitoba	6.0% (289)	5.6% (258)	5.3% (308)	3.7%	3.4%	3.8%
Ontario	23.4% (1,134)	20.6% (959)	33.3% (1,932)	41.1%	40.6%	40.6%
Quebec	18.4% (892)	13.5% (626)	12.6% (729)	17.5%	17.6%	17.4%
New Brunswick	5.1% (246)	7.1% (332)	5.6% (322)	2.3%	2.4%	2.3%
Nova Scotia	6.8% (328)	7.1% (328)	6.1% (355)	3.1%	3.0%	2.9%
Prince Edward Island	0.8% (41)	1.3% (62)	1.1% (65)	0.4%	0.4%	0.4%
Newfoundland and Labrador	3.4% (167)	6.1% (284)	3.8% (220)	1.4%	1.6%	1.4%
Urban/rural						
Rural	12.3% (501)	13.5% (538)	13.1% (651)	11.4%	10.1%	88.1%
Urban	87.7% (3,573)	86.5% (3,453)	86.9% (4,320)	88.6%	89.9%	11.9%

### Euclidean distance to nearest legal retail store

**Figure 1.**
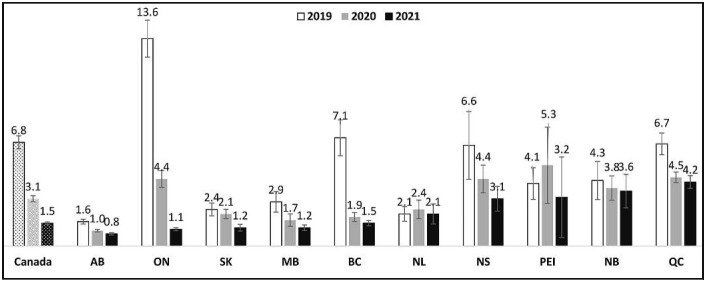
Median Euclidean distance to the nearest legal retail store (km) among past-12-month cannabis consumers of provincial legal age to purchase cannabis, 2019–2021 (*n* = 13,062). Units are kilometers (1 km = 0.62 miles). Provinces ordered smallest to largest median in 2021. AB = Alberta; ON = Ontario; SK = Saskatchewan; MB = Manitoba; BC = British Columbia; NL = Newfoundland and Labrador; NS = Nova Scotia; PEI = Prince Edward Island; NB = New Brunswick; QC = Québec.

Figure 1 displays the median Euclidean distance to the nearest legal retail stores among respondents in the study sample. Across all 10 provinces, the median distance to the nearest legal retail store was 6.8 km in 2019, 3.1 km in 2020, and 1.5 km in 2021. The median distance ranged from 1.6 km in Alberta to 13.6 km in Ontario in 2019, from 1.0 km in Alberta to 5.3 km in Prince Edward Island in 2020, and from 0.8 km in Alberta to 4.2 km in Québec in 2021.

Table 2 displays seven regression models examining the correlates of cannabis sources used in the past 12 months. Respondents living less than 10 km from their nearest legal retail store had lower odds of growing their own cannabis versus respondents living more than 10 km away. Respondents living less than 3 km from their nearest legal retail store had lower odds of obtaining cannabis from a legal website versus respondents living more than 10 km away. Respondents living less than 3 km from their nearest legal retail store had higher odds of obtaining cannabis from a legal store versus respondents living 10 km away or more.

**Table 2.**
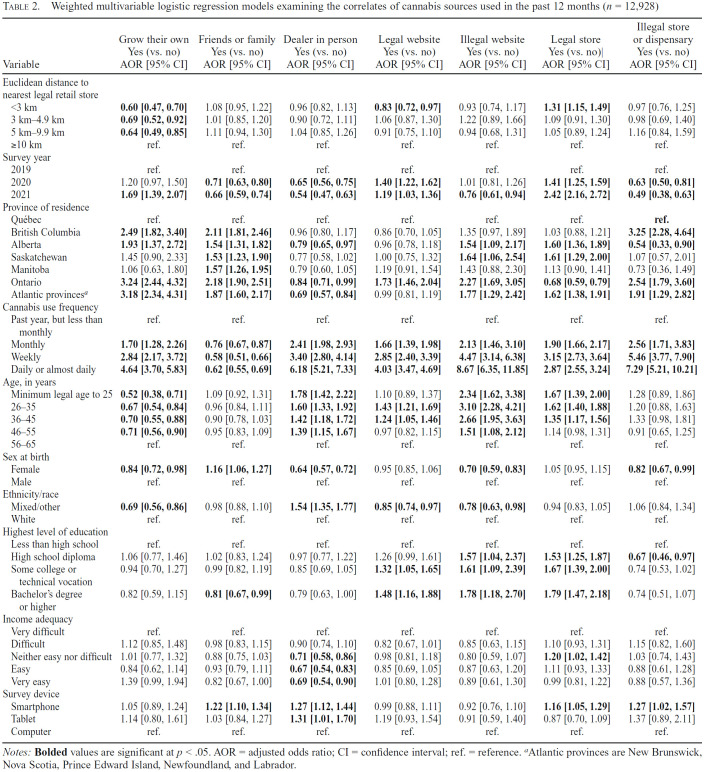
Weighted multivariable logistic regression models examining the correlates of cannabis sources used in the past 12 months (*n* = 12,928)

Variable	Grow their own Yes (vs. no) AOR [95% CI]	Friends or family Yes (vs. no) AOR [95% CI]	Dealer in person Yes (vs. no) AOR [95% CI]	Legal website Yes (vs. no) AOR [95% CI]	Illegal website Yes (vs. no) AOR [95% CI]	Legal store Yes (vs. no)| AOR [95% CI]	Illegal store or dispensary Yes (vs. no) AOR [95% CI]
Euclidean distance to nearest legal retail store							
<3 km	**0.60 [0.47, 0.70]**	1.08 [0.95, 1.22]	0.96 [0.82, 1.13]	**0.83 [0.72, 0.97]**	0.93 [0.74, 1.17]	**1.31 [1.15, 1.49]**	0.97 [0.76, 1.25]
3 km-4.9km	**0.69 [0.52, 0.92]**	1.01 [0.85, 1.20]	0.90 [0.72, 1.11]	1.06 [0.87, 1.30]	1.22 [0.89, 1.66]	1.09 [0.91, 1.30]	0.98 [0.69, 1.40]
5 km-9.9km	**0.64 [0.49, 0.85]**	1.11 [0.94, 1.30]	1.04 [0.85, 1.26]	0.91 [0.75, 1.10]	0.94 [0.68, 1.31]	1.05 [0.89, 1.24]	1.16 [0.84, 1.59]
≥10 km	ref.	ref.	ref.	ref.	ref.	ref.	ref.
Survey year							
2019	ref.	ref.	ref.	ref.	ref.	ref.	ref.
2020	1.20 [0.97, 1.50]	**0.71 [0.63, 0.80]**	**0.65 [0.56, 0.75]**	**1.40 [1.22, 1.62]**	1.01 [0.81, 1.26]	**1.41 [1.25, 1.59]**	**0.63 [0.50, 0.81]**
2021	**1.69 [1.39, 2.07]**	**0.66 [0.59, 0.74]**	**0.54 [0.47, 0.63]**	**1.19 [1.03, 1.36]**	**0.76 [0.61, 0.94]**	**2.42 [2.16, 2.72]**	**0.49 [0.38, 0.63]**
Province of residence							
Québec	ref.	ref.	ref.	ref.	ref.	ref.	**ref.**
British Columbia	**2.49 [1.82, 3.40]**	**2.11 [1.81, 2.46]**	0.96 [0.80, 1.17]	0.86 [0.70, 1.05]	1.35 [0.97, 1.89]	1.03 [0.88, 1.21]	**3.25 [2.28, 4.64]**
Alberta	**1.93 [1.37, 2.72]**	**1.54 [1.31, 1.82]**	**0.79 [0.65, 0.97]**	0.96 [0.78, 1.18]	**1.54 [1.09, 2.17]**	**1.60 [1.36, 1.89]**	**0.54 [0.33, 0.90]**
Saskatchewan	1.45 [0.90, 2.33]	**1.53 [1.23, 1.90]**	0.77 [0.58, 1.02]	1.00 [0.75, 1.32]	**1.64 [1.06, 2.54]**	**1.61 [1.29, 2.00]**	1.07 [0.57, 2.01]
Manitoba	1.06 [0.63, 1.80]	**1.57 [1.26, 1.95]**	0.79 [0.60,1.05]	1.19 [0.91, 1.54]	1.43 [0.88, 2.30]	1.13 [0.90, 1.41]	0.73 [0.36, 1.49]
Ontario	**3.24 [2.44, 4.32]**	**2.18 [1.90, 2.51]**	**0.84 [0.71, 0.99]**	**1.73 [1.46, 2.04]**	**2.27 [1.69, 3.05]**	**0.68 [0.59, 0.79]**	**2.54 [1.79, 3.60]**
Atlantic provinces^a^	**3.18 [2.34, 4.31]**	**1.87 [1.60, 2.17]**	**0.69 [0.57, 0.84]**	0.99 [0.81, 1.19]	**1.77 [1.29, 2.42]**	**1.62 [1.38, 1.91]**	**1.91 [1.29, 2.82]**
Cannabis use frequency							
Past year, but less than monthly	ref.	ref.	ref.	ref.	ref.	ref.	ref.
Monthly	**1.70 [1.28, 2.26]**	**0.76 [0.67, 0.87]**	**2.41 [1.98, 2.93]**	**1.66 [1.39, 1.98]**	**2.13 [1.46, 3.10]**	**1.90 [1.66, 2.17]**	**2.56 [1.71, 3.83]**
Weekly	**2.84 [2.17, 3.72]**	**0.58 [0.51, 0.66]**	**3.40 [2.80, 4.14]**	**2.85 [2.40, 3.39]**	**4.47 [3.14, 6.38]**	**3.15 [2.73, 3.64]**	**5.46 [3.77, 7.90]**
Daily or almost daily	**4.64 [3.70, 5.83]**	**0.62 [0.55, 0.69]**	**6.18 [5.21, 7.33]**	**4.03 [3.47, 4.69]**	**8.67 [6.35, 11.85]**	**2.87 [2.55, 3.24]**	**7.29 [5.21, 10.21]**
Age, in years							
Minimum legal age to 25	**0.52 [0.38, 0.71]**	1.09 [0.92, 1.31]	**1.78 [1.42, 2.22]**	1.10 [0.89, 1.37]	**2.34 [1.62, 3.38]**	**1.67 [1.39, 2.00]**	1.28 [0.89, 1.86]
26-33	**0.67 [0.54, 0.84]**	0.96 [0.84, 1.11]	**1.60 [1.33, 1.92]**	**1.43 [1.21, 1.69]**	**3.10 [2.28, 4.21]**	**1.62 [1.40, 1.88]**	1.20 [0.88, 1.63]
36-45	**0.70 [0.55, 0.88]**	0.90 [0.78, 1.03]	**1.42 [1.18, 1.72]**	**1.24 [1.05, 1.46]**	**2.66 [1.95, 3.63]**	**1.35 [1.17, 1.56]**	1.33 [0.98, 1.81]
46-55	**0.71 [0.56, 0.90]**	0.95 [0.83, 1.09]	**1.39 [1.15, 1.67]**	0.97 [0.82, 1.15]	**1.51 [1.08, 2.12]**	1.14 [0.98, 1.31]	0.91 [0.65, 1.25]
56-65	ref.	ref.	ref.	ref.	ref.	ref.	ref.
Sex at birth							
Female	**0.84 [0.72, 0.98]**	**1.16 [1.06, 1.27]**	**0.64 [0.57, 0.72]**	0.95 [0.85, 1.06]	**0.70 [0.59, 0.83]**	1.05 [0.95, 1.15]	**0.82 [0.67, 0.99]**
Male	ref.	ref.	ref.	ref.	ref.	ref.	ref.
Ethnicity/race							
Mixed/other	**0.69 [0.56, 0.86]**	0.98 [0.88, 1.10]	**1.54 [1.35, 1.77]**	**0.85 [0.74, 0.97]**	**0.78 [0.63, 0.98]**	0.94 [0.83, 1.05]	1.06 [0.84, 1.34]
White	ref.	ref.	ref.	ref.	ref.	ref.	ref.
Highest level of education							
Less than high school	ref.	ref.	ref.	ref.	ref.	ref.	ref.
High school diploma	1.06 [0.77, 1.46]	1.02 [0.83, 1.24]	0.97 [0.77, 1.22]	1.26 [0.99, 1.61]	**1.57 [1.04, 2.37]**	**1.53 [1.25, 1.87]**	**0.67 [0.46, 0.97]**
Some college or technical vocation	0.94 [0.70, 1.27]	0.99 [0.82, 1.19]	0.85 [0.69, 1.05]	**1.32 [1.05, 1.65]**	**1.61 [1.09, 2.39]**	**1.67 [1.39, 2.00]**	0.74 [0.53, 1.02]
Bachelor's degree or higher	0.82 [0.59,1.15]	**0.81 [0.67, 0.99]**	0.79 [0.63, 1.00]	**1.48 [1.16, 1.88]**	**1.78 [1.18, 2.70]**	**1.79[1.47, 2.18]**	0.74 [0.51, 1.07]
Income adequacy							
Very difficult	ref.	ref.	ref.	ref.	ref.	ref.	ref.
Difficult	1.12 [0.85, 1.48]	0.98 [0.83, 1.15]	0.90 [0.74, 1.10]	0.82 [0.67, 1.01]	0.85 [0.63, 1.15]	1.10 [0.93, 1.31]	1.15 [0.82, 1.60]
Neither easy nor difficult	1.01 [0.77, 1.32]	0.88 [0.75, 1.03]	**0.71 [0.58, 0.86]**	0.98 [0.81, 1.18]	0.80 [0.59, 1.07]	**1.20 [1.02, 1.42]**	1.03 [0.74, 1.43]
Easy	0.84 [0.62, 1.14]	0.93 [0.79, 1.11]	**0.67 [0.54, 0.83]**	0.85 [0.69, 1.05]	0.87 [0.63, 1.20]	1.11 [0.93, 1.33]	0.88 [0.61, 1.28]
Very easy	1.39 [0.99, 1.94]	0.82 [0.67, 1.00]	**0.69 [0.54, 0.90]**	1.01 [0.80, 1.28]	0.89 [0.61, 1.30]	0.99 [0.81, 1.22]	0.88 [0.57, 1.36]
Survey device							
Smartphone	1.05 [0.89, 1.24]	**1.22 [1.10, 1.34]**	**1.27 [1.12, 1.44]**	0.99 [0.88, 1.11]	0.92 [0.76, 1.10]	**1.16 [1.05, 1.29]**	**1.27 [1.02, 1.57]**
Tablet	1.14 [0.80, 1.61]	1.03 [0.84, 1.27]	**1.31 [1.01, 1.70]**	1.19 [0.93, 1.54]	0.91 [0.59, 1.40]	0.87 [0.70, 1.09]	1.37 [0.89, 2.11]
Computer	ref.	ref.	ref.	ref.	ref.	ref.	ref.

*Notes:* Bolded values are significant at *p* < .05. AOR = adjusted odds ratio; CI = confidence interval; ref. = reference.

^a^ Atlantic provinces are New Brunswick, Nova Scotia, Prince Edward Island, Newfoundland, and Labrador.

In a sensitivity analysis, all models were refitted to include respondents who did not provide a postal code and similar patterns emerged (Supplemental Table A). Respondents who did not provide postal code data had lower odds of obtaining cannabis from friends or family, legal and illegal websites, and illegal stores or dispensaries.

### Survey year

**Figure 2.**
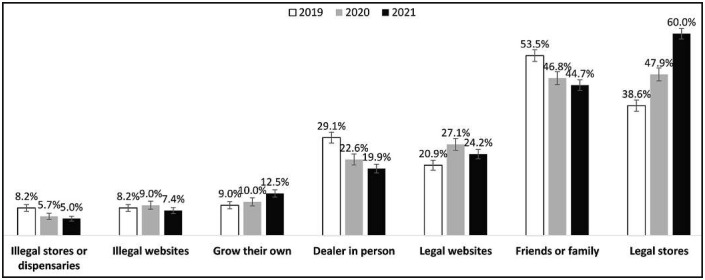
Sources used to obtain cannabis in the past 12 months among past-12-month cannabis consumers of provincial legal age to purchase cannabis, 2019–2021 (*n* = 15,311). Respondents could select all that applied. Percentages are weighted.

Figure 2 displays the sources used to obtain cannabis in the past 12 months in 2019–2021. In 2019, the most common source was friends or family. In 2020 and 2021, the most common source was legal retail stores.

Respondents in 2021 had higher odds of growing their own cannabis versus respondents in 2019 (Table 2). Respondents in 2020 and 2021 had lower odds of obtaining cannabis from dealers in person or family and friends versus respondents in 2019. Respondents in 2020 and 2021 had higher odds of obtaining cannabis from legal websites or legal stores versus respondents in 2019. Respondents in 2021 had lower odds of obtaining cannabis from illegal websites versus respondents in 2019. Respondents in 2020 and 2021 had lower odds of obtaining cannabis from illegal stores or dispensaries versus respondents in 2019.

Interactions between survey year and distance to nearest legal retail store were not statistically significant (grew their own: *F* = 1.1, *p* = .377; friends or family: *F* = 1.2, *p* = .317; dealer in person: *F* = 1.8, *p* = .087; legal websites: *F* = 1.4, *p* = .197; illegal websites: *F* = 0.6, *p* = .703; legal stores: *F* = 1.1, *p* = .352; illegal stores or dispensaries: *F* = 0.4, *p* = .871).

### Province of residence

Respondents in all provinces except Saskatchewan and Manitoba had higher odds of growing their own cannabis versus respondents in Québec (Table 2). Respondents in all other provinces had higher odds of obtaining cannabis from family and friends versus respondents in Québec. Respondents in Alberta, Ontario, and the Atlantic provinces had lower odds of obtaining cannabis from a dealer versus respondents in Québec. Respondents in Ontario had higher odds of obtaining cannabis from a legal website versus respondents in Québec. Respondents in Alberta, Saskatchewan, and the Atlantic provinces had higher odds of obtaining cannabis from illegal websites and legal stores versus Québec. Respondents in Ontario had lower odds of obtaining cannabis from legal stores versus Québec. Respondents in British Columbia, Ontario, and the Atlantic provinces had higher odds of obtaining cannabis from illegal stores or dispensaries versus Québec. Respondents in Alberta had lower odds of obtaining cannabis from illegal stores or dispensaries versus respondents in Québec.

## Discussion

The current study is the first to examine the distance to the nearest legal retail store and its relationship to the sources used to obtain cannabis in the Canadian cannabis market since legalization. The study has four primary findings: First, on average, respondents lived closer to a legal retail store in 2021 than in 2019. Second, as of 2020, legal retail stores surpassed friends and family as the most commonly used source to obtain cannabis in the past 12 months. Third, respondents living closest to a legal store were more likely to source from legal stores and less likely to grow their own cannabis or source from legal websites relative to those living further from a legal store. Fourth, a greater percentage of respondents sourced their cannabis from legal sources (growing their own, legal stores, legal websites), and a lower percentage of respondents sourced their cannabis from illegal sources (dealers, illegal stores, illegal websites) in 2021 than in 2019.

On average, Canadian cannabis consumers lived closer to a legal retail store in 2021 than they did in 2019. The median distance to the nearest legal retail store across the 10 provinces was 1.5 km in 2021 compared with 6.8 km in 2019.

The reduction in average distance can be explained by the substantial increase in the number of legal stores that opened between 2019 and 2021, especially in Ontario, Canada's most populous province (Alcohol and Gaming Commission of Ontario, 2021; Myran et al., 2022). In September 2019 (to align with the ICPS 2019 survey date), Ontario had 24 retail stores, which increased to 1,042 stores in September 2021. Indeed, this was reflected in the current study, where the average median distance to a nearest store in Ontario decreased from 14 km to 1 km in 2 years. The number of retail stores has continued to increase through 2022, although closures of some stores has also occurred because of competition (Labby, 2022; Saba, 2022).

The average distance from a legal retail store varied across the provinces. In 2021, respondents in Alberta were the closest to a legal retail store, and respondents in Québec were the furthest. Alberta had 18.7 stores per 100,000 residents age 15 and older compared with Québec, with 0.8 stores per 100,000 residents in 2021 (Alberta Gaming, Liquor and Cannabis, 2022; Statistics Canada, 2020). Respondents from Alberta were more likely to source from legal retail stores and less likely to source from illegal stores or dispensaries than those in Québec, perhaps because of the contrast in accessibility and availability of legal retail stores. However, the relative difference in retail stores per capita between Alberta and Québec—more than 23-fold higher in Alberta—was substantially greater than the difference in sourcing from legal stores, where respondents in Alberta were only 1.6 times more likely to source from legal stores than those in Québec. Moreover, respondents from Ontario, with 8.3 stores per 100,000 residents age 15 and older, were less likely to source from legal stores compared with respondents from Québec. This suggests that the association between retail stores per capita and sourcing from legal retail stores is not linear or immediate and may have diminishing returns as the number of retail stores reaches higher levels.

Over the 3 years, approximately half of respondents obtained cannabis from friends and family. However, a smaller percentage of respondents sourced from family and friends; by 2020, legal stores had surpassed friends and family as the most popular source of cannabis. In fact, a greater percentage of respondents obtained cannabis from legal sources (e.g., legal stores and websites) over time, whereas a smaller percentage obtained from illegal sources (e.g., dealers, illegal websites, and dispensaries) over time, indicating a general shift to the legal market. These results mirror those reported in the Canadian Cannabis Survey, in which the percentage of respondents reporting that they “usually” source from friends and family and dealers decreased from 2019 to 2021, whereas those reporting that they usually sourced from legal storefronts increased (Health Canada, 2019, 2021).

Use of legal cannabis websites increased in 2020 relative to 2019 but declined in 2021. Respondents were more likely to source cannabis online in 2020, which may be explained by the COVID-19 pandemic and the timing of our survey: Data collection began in September 2020, approximately 6 months after lockdowns began. In Canada, online sales are legal in all provinces and territories. Except for some provinces that temporarily declared cannabis stores to be “essential services,” stores were closed during lockdowns, so legal cannabis was available only online (Canadian Centre on Substance Use and Addiction, 2021). However, curbside pickup was introduced shortly afterward, enabling consumers to collect from the physical retail stores, which might potentially explain the lack of sustained increase in online sources beyond 2020 (Canadian Centre on Substance Use and Addiction, 2021).

Household proximity to a legal retail store was also associated with sourcing cannabis from both legal online and physical stores, but only among those who lived very close (<3 km). These results may reflect accessibility and convenience for those living within walking distance or a short drive from a store. Distance to the nearest legal store was not associated with sourcing from friends or family, dealers, illegal websites, and illegal stores. The lack of association may be attributable to these relationships being established before legalization. Proximity to legal retail stores may not be the driving factor encouraging respondents to transition to the legal market. It may be that living closer to a legal store doesn't necessarily transition consumers away from illegal sources but merely provides more options of where to source. This is an important finding in light of the intended balance between promoting market transition and promoting consumption. Indeed, although the literature on retail proximity and cannabis use is mixed (Everson et al., 2019; Hust et al., 2020; Palali & van Ours, 2015; Shi, 2016; Shi et al., 2018), literature in the alcohol and tobacco field suggests that living close to retail stores increases use (Freisthler et al., 2003; Lipperman-Kreda et al., 2012, 2014; U.S. National Cancer Institute and World Health Organization, 2016). To achieve public health outcomes, provincial and federal governments must ensure that increased access does not increase consumption or initiation, especially among young people.

Provincial policies appeared to influence whether consumers grew their own cannabis. Respondents in all provinces except Manitoba and Saskatchewan were more likely to grow or make their own cannabis than those in Québec. Manitoba and Québec are the only provinces to prohibit home cultivation, which may explain their lower cultivation rates. Indeed, in a previous study from the ICPS, residents from Québec and Manitoba were less likely to grow their own cannabis than residents of all other provinces (Wadsworth et al., 2022). Québec also has had the lowest prices of cannabis, which may negate the cost-benefits of growing or making one's own cannabis (Gibbs et al., 2021; Mahamad & Hammond, 2019; Mahamad et al., 2020; Martin, 2019; Wadsworth et al., 2020).

### Limitations

This study is subject to limitations common to survey research. Respondents were recruited using non–probability-based sampling; therefore, the findings do not necessarily provide nationally representative estimates. The data were weighted by age group, sex, region, education, and tobacco smoking status in Canada. ICPS cannabis use estimates were generally lower than national estimates for young adults and higher than national surveys in Canada (Health Canada, 2019, 2020, 2021). This is likely because the ICPS sampled individuals ages 16–65, whereas national surveys included older adults, who are known to have lower rates of cannabis use. However, this limitation may have only a modest effect on understanding purchasing patterns relative to proximity to cannabis stores among people who currently use cannabis.

Euclidean distance to legal retail cannabis stores was treated as a categorical variable in the regression models, which assumes there are similar break points in distance traveled among respondents. A continuous measure would assume a monotonic linear relationship between distance and sourcing cannabis. Moreover, the geometric mean revealed skewness in the data and so a continuous measure was deemed inappropriate. Sensitivity analyses were conducted previously to examine the effect of distance as a categorical measure using different classification schemes as well as a continuous measure and similar patterns emerged (Wadsworth et al., 2021).

There were systematic differences between those who provided a postal code and those who did not. Respondents who provided a postal code were more likely to be older, well educated, and of White ethnicity/race, and reported making ends meet easy or difficult, which may have influenced where respondents lived and therefore our results and interpretations. However, sensitivity analyses were conducted including those who did not provide a postal code in the models and similar patterns emerged in all models (Supplemental Table A).

### Conclusions

Canadians of legal age to purchase cannabis appear to be transitioning to the legal market. In the 3 years since legalization in Canada, the use of traditional and illegal sources of cannabis decreased over time, whereas use of legal stores increased over time. In 2021, the study sample lived closer to a legal retail store than in 2020 and 2019, due to more stores being open in 2021. Household proximity to a legal retail store, measured in Euclidean distance, significantly contributed to the use of some sources but not all: Distance did not matter with regard to sourcing from dealers, family or friends, or illegal websites/stores but did matter with sourcing in legal stores, legal websites, or when growing cannabis. Overall, the findings depict that proximity to legal cannabis stores may aid uptake of the legal market. However, there may be diminishing returns at a certain point; indeed, proximity was not associated with illegal sources. This may indicate that using a conservative proximity level to avoid promotion of use may have greater public health impact than increasing density in the interest of promoting transition to legal sources.

## Conflict-of-Interest Statement

The authors report no conflicts of interest. DH has served as a paid Expert Witness on behalf of public health authorities in response to industry legal challenges to cannabis regulations in Canada.

## References

[B1] Alberta Gaming, Liquor and Cannabis 2022 Cannabis licensee search https://aglc.ca/cannabis/retail-cannabis/cannabis-licensee-search

[B2] Alcohol and Gaming Commission of Ontario 2021 The AGCO now issuing 30 cannabis retail store authorizations per week https://www.agco.ca/blog/cannabis/feb-2021/agco-now-issuing-30-cannabis-retail-store-authorizations-week

[B3] Alcohol and Gaming Commission of Ontario 2022 List of Ontario municipalities prohibiting or allowing cannabis retail stores https://www.agco.ca/cannabis/list-ontario-municipalities-prohibiting-or-allowing-cannabis-retail-stores?title_field_value=&field_opt_in_out_value_i18n=All&order=field_opt_in_out&sort=asc&page=18

[B4] American Association for Public Opinion Research 2016 Standard definitions: Final dispositions of case codes and outcome rates for surveys Retrieved January 30, 2021, from https://www.aapor.org/AAPOR_Main/media/publications/Standard-Definitions20169theditionfinal.pdf

[B5] Amiri S. Monsivais P. McDonell M. G. Amram O. 2019 Availability of licensed cannabis businesses in relation to area deprivation in Washington state: A spatiotemporal analysis of cannabis business presence between 2014 and 2017 Drug and Alcohol Review 38 7 790 797 10.1111/dar.12987 31647158

[B6] Armstrong M. J. 2021 Relationships between increases in Canadian cannabis stores, sales, and prevalence Drug and Alcohol Dependence 228 109071 10.1016/j.drugalcdep.2021.109071 34592703

[B7] Armstrong M. J. Cantor N. Smith B. T. Jesseman R. Hobin E. Myran D. T. 2022 Interrupted time series analysis of Canadian legal cannabis sales during the COVID-19 pandemic Drug and Alcohol Review 41 5 1131 1135 10.1111/dar.13465 35316855 PMC9111650

[B8] Azofeifa A. Pacula R. L. Mattson M. E. 2021 Cannabis growers in the United States: Findings from a National Household Survey 2010−2014 Journal of Drug Issues 51 3 518 530 10.1177/00220426211000457

[B9] Bow C. J. D. Waters N. M. Faris P. D. Seidel J. E. Galbraith P. D. Knudtson M. L. Ghali W. A. The APPROACH Investigators 2004 Accuracy of city postal code coordinates as a proxy for location of residence International Journal of Health Geographics 3 1 5 10.1186/1476-072X-3-5 15028120 PMC394341

[B10] Canadian Centre on Substance Use and Addiction 2020 Policy and regulations (Cannabis) https://www.ccsa.ca/policy-and-regulations-cannabis

[B11] Canadian Centre on Substance Use and Addiction 2021 Cannabis retail during COVID-19: A policy brief https://www.ccsa.ca/sites/default/files/2021-01/CCSA-COVID-19-Cannabis-Retail-Policy-Brief-2021-en.pdf

[B12] Corsetti D. Burkhalter R. Hammond D. 2022 International Cannabis Policy Study Technical Report – Wave 4 (2021) University of Waterloo http://cannabisproject.ca/methods/

[B13] Department of Cannabis Control 2022 Where cannabis businesses are allowed https://cannabis.ca.gov/cannabis-laws/where-cannabis-businesses-are-allowed/

[B14] Dilley J. A. Hitchcock L. McGroder N. Greto L. A. Richardson S. M. 2017 Community-level policy responses to state marijuana legalization in Washington State International Journal on Drug Policy 42 102 108 10.1016/j.drugpo.2017.02.010 28365192 PMC5473373

[B15] Everson E. M. Dilley J. A. Maher J. E. Mack C. E. 2019 Post-legalization opening of retail cannabis stores and adult cannabis use in Washington State, 2009-2016 American Journal of Public Health 109 9 1294 1301 10.2105/AJPH.2019.305191 31318588 PMC6687243

[B16] Firth C. L. Carlini B. Dilley J. Guttmannova K. Hajat A. 2022 Retail cannabis environment and adolescent use: The role of advertising and retailers near home and school Health & Place 75 102795 10.1016/j.healthplace.2022.102795 35344691 PMC9189000

[B17] Firth C. L. Carlini B. H. Dilley J. A. Wakefield J. Hajat A. 2020 What about equity? Neighborhood deprivation and cannabis retailers in Portland, Oregon Cannabis 3 2 157 172 10.26828/cannabis.2020.02.003

[B18] Freisthler B. Gruenewald P. J. Treno A. J. Lee J. 2003 Evaluating alcohol access and the alcohol environment in neighborhood areas Alcoholism: Clinical and Experimental Research 27 3 477 484 10.1097/01.ALC.0000057043.04199.B7 12658114

[B19] Gibbs B. Reed T. Wride S. 2021 Cannabis legalisation – Canada's experience. A research report by Public First https://www.publicfirst.co.uk/wp-content/uploads/2021/10/REPORT-Cannabis-in-Canada-Public-First-October-2021.pdf

[B20] Goodman S. Burkhalter R. Hammond D. 2020 International Cannabis Policy Study Technical Report –Wave 2 (2019 University of Waterloo http://cannabisproject.ca/methods/

[B21] Goodman S. Burkhalter R. Hammond D. 2021 International Cannabis Policy Study Technical Report Wave 3 2020 University of Waterloo http://cannabisproject.ca/methods/

[B22] Government of Canada 2022 Cannabis use for non-medical purposes among Canadians (aged 16+) https://health-infobase.canada.ca/cannabis/

[B23] Hammond D. Goodman S. Wadsworth E. Rynard V. Boudreau C. Hall W. 2020 Evaluating the impacts of cannabis legalization: The International Cannabis Policy Study International Journal on Drug Policy 77 102698 10.1016/j.drugpo.2020.102698 32113149

[B24] Canada Health 2019 Canadian Cannabis Survey 2019: Summary https://www.canada.ca/en/health-canada/services/publications/drugs-health-products/canadian-cannabis-survey-2019-summary.html

[B25] Canada Health 2021 Canadian Cannabis Survey 2021: Summary https://www.canada.ca/en/health-canada/services/drugs-medication/cannabis/research-data/canadian-cannabis-survey-2021-summary.html#a1

[B26] Hust S. J. T. Willoughby J. F. Li J. Couto L. 2020 Youth's proximity to marijuana retailers and advertisements: Factors associated with Washington State adolescents’ intentions to use marijuana Journal of Health Communication 25 7 594 603 10.1080/10810730.2020.1825568 33030100

[B27] Labby B. 2022 September 12 The number of pot stores in Alberta reaches a potentially unsustainable high CBC News https://www.cbc.ca/news/canada/calgary/alberta-cannabis-retail-oversaturation-1.6577520

[B28] Leafly Canada Staff 2019 November 25 Ontario Cannabis Store launches same-day delivery https://www.leafly.com/news/industry/ocs-same-day-delivery

[B29] Lipperman-Kreda S. Grube J. W. Friend K. B. 2012 Local tobacco policy and tobacco outlet density: Associations with youth smoking Journal of Adolescent Health 50 547 552 10.1016/j.jadohealth.2011.08.015 PMC3360878 22626479

[B30] Lipperman-Kreda S. Mair C. Grube J. W. Friend K. B. Jackson P. Watson D. 2014 Density and proximity of tobacco outlets to homes and schools: Relations with youth cigarette smoking Prevention Science 15 5 738 744 10.1007/s11121-013-0442-2 24254336 PMC4029873

[B31] Mahamad S. Hammond D. 2019 Retail price and availability of illicit cannabis in Canada Addictive Behaviors 90 402 408 10.1016/j.addbeh.2018.12.001 30530299

[B32] Mahamad S. Wadsworth E. Rynard V. Goodman S. Hammond D. 2020 Availability, retail price and potency of legal and illegal cannabis in Canada after recreational cannabis legalisation Drug and Alcohol Review 39 4 337 346 10.1111/dar.13069 32291811

[B33] Martin B. 2019 Price averages for Canadian cannabis by province Leafly Retrieved February 23, 2021, from https://www.leafly.ca/news/strains-products/canada-price-averages

[B34] Myran D. T. Staykov E. Cantor N. Taljaard M. Quach B. I. Hawken S. Tanuseputro P. 2022 How has access to legal cannabis changed over time? An analysis of the cannabis retail market in Canada 2years following the legalisation of recreational cannabis Drug and Alcohol Review 41 2 377 385 10.1111/dar.13351 34250645

[B35] Oregon Liquor and Cannabis Commission 2022 Record of cities/counties prohibiting licensed recreational marijuana facilities 2022 (revised 7/13/2022) https://www.oregon.gov/olcc/marijuana/Documents/Cities_Counties_RMJOptOut.pdf

[B36] Palali A. van Ours J. C. 2015 Distance to cannabis shops and age of onset of cannabis use Health Economics 24 11 1483 1501 10.1002/hec.3104 25294622

[B37] Paschall M. J. Grube J. W. 2020 Recreational marijuana availability in Oregon and use among adolescents American Journal of Preventive Medicine 58 2 e63 e69 10.1016/j.amepre.2019.09.020 31959327 PMC6986306

[B38] Pinault L. Khan S. Tjepkema M. 2020 June 17 Accuracy of matching residential postal codes to census geography Health Reports 31 3 3 13 10.25318/82-003-x202000300001-eng 32644759

[B39] Popova S. Giesbrecht N. Bekmuradov D. Patra J. 2009 Hours and days of sale and density of alcohol outlets: Impacts on alcohol consumption and damage: A systematic review Alcohol and Alcoholism 44 5 500 516 10.1093/alcalc/agp054 19734159

[B40] Saba R. 2022 September 3 Toronto's pot shop reckoning is here — industry watchers predict up to a third of our stores will close The Toronto Star https://www.thestar.com/business/2022/09/03/torontospot-shop-reckoning-is-here-industry-watchers-predict-up-to-a-third-ofour-stores-will-close.html

[B41] Shi Y. 2016 The availability of medical marijuana dispensary and adolescent marijuana use Preventive Medicine 91 1 7 10.1016/j.ypmed.2016.07.015 27471020

[B42] Shi Y. Cummins S. E. Zhu S. H. 2018 Medical marijuana availability, price, and product variety, and adolescents’ marijuana use Journal of Adolescent Health 63 1 88 93 10.1016/j.jadohealth.2018.01.008 PMC6070346 30060862

[B43] Shi Y. Meseck K. Jankowska M. M. 2016 Availability of medical and recreational marijuana stores and neighborhood characteristics in Colorado Journal of Addiction 2016 7193740 10.1155/2016/7193740 27213075 PMC4860233

[B44] Statistics Canada 2019 Analysis in brief: The retail cannabis market in Canada, a portrait of the first year https://www150.statcan.gc.ca/n1/pub/11-621-m/11-621-m2019005-eng.htm

[B45] Statistics Canada 2020 Estimates of population as of July 1st, by marital status or legal marital status, age and sex Table: 17-10-0060-01 (formerly CANSIM 051-0042). Retrieved March 23, 2021, from https://www150.statcan.gc.ca/t1/tbl1/en/tv.action?pid=1710006001

[B46] Statistics Canada 2021 Postal Code Conversion File Plus (PCCF+) Version 7D Reference Guide: November 2020 Postal Codes https://abacus.library.ubc.ca/dataset.xhtml?persistentId=hdl:11272.1/AB2/TVD98Q

[B47] Statistics Canada 2022 Table 13-10-0096-10. Smokers, by age group 10.25318/1310009601-eng

[B48] Tabb L. P. Fillmore C. Melly S. 2018 Location, location, location: Assessing the spatial patterning between marijuana licenses, alcohol outlets and neighborhood characteristics within Washington state Drug and Alcohol Dependence 185 214 218 10.1016/j.drugalcdep.2018.01.004 29471225

[B49] Unger J. B. Vos R. O. Wu J. S. Hardaway K. Sarain A. Y. L. Soto D. W. Rogers C. Steinberg J. 2020 Locations of licensed and unlicensed cannabis retailers in California: A threat to health equity? Preventive Medicine Reports 19 101165 10.1016/j.pmedr.2020.101165 32714779 PMC7378688

[B50] National U.S. Cancer Institute and World Health Organization 2016 The economics of tobacco and tobacco control. National Cancer Institute Tobacco Control Monograph 21 NIH Publication No. 16-CA-8029A https://cancercontrol.cancer.gov/brp/tcrb/monographs/monograph-21

[B51] Wadsworth E. Cristiano N. Pacheco K. Jesseman R. Hammond D. 2022 Home cultivation across Canadian provinces after cannabis legalization Addictive Behaviors Reports 15 100423 10.1016/j.abrep.2022.100423 35434251 PMC9006643

[B52] Wadsworth E. Driezen P. Goodman S. Hammond D. 2020 Differences in self-reported cannabis prices across purchase source and quantity purchased among Canadians Addiction Research and Theory 28 6 474 483 10.1080/16066359.2019.1689961

[B53] Wadsworth E. Driezen P. Hammond D. 2021 Retail availability and legal purchases of dried flower in Canada post-legalization Drug and Alcohol Dependence 225 108794 10.1016/j.drugalcdep.2021.108794 34098382

